# Supplementary material for: Understanding biochemical design principles with ensembles of canonical non-linear models
Source: PLoS One. 2020 Apr 30;15(4):e0230599. doi: 10.1371/journal.pone.0230599 (PMC7192416; doi:10.1371/journal.pone.0230599)
Supplement: S1 Appendix — (PDF) [file pone.0230599.s001.pdf]

1 Supplementary information

1.1 Estimation of a realistic flux

To illustrate the example using a reasonable value for the flux through a biosynthetic pathway, the reference was chosen of a bacteria growing with a duplication time of 20 min ($\mu = 0.035\,\mathrm{min}^{-1}$) and having a protein content of 55%. Assuming that a certain aminoacid has an abundance of 5%, that would result in a flux of 0.0087 milimoles per minute and gram of dry weight. Since dry weight is about 30% of the biomass, which has a density 1.1 times that of water, the flux in molar concentration is approximately 30 mM /min.

1.2 GMA and s-systems

The mass balance:

$$\dot{\mathbf{x}}_{\mathbf{d}} = \mathbf{N}\,\mathbf{v}(\mathbf{x}_{\mathbf{d}}(t), \mathbf{x}_{\mathbf{i}}) \tag{1}$$

where $\mathbf{N}$, the stoichiometric matrix, contains all the relevant information on the structure of the fluxes within the network and the vector of reaction rates, $\mathbf{v}$. The vector can be partitioned in a set of independent variable x_i and dependent variables x_d. The vector of reaction rates $\mathbf{v}$ can represent any rate law. In Biochemical Systems Theory (BST), a power-law expression is used for each flux:

$$v_i = \gamma_i \prod_{j=1}^{n+m} x_j^{f_{i,j}} \tag{2}$$

where, by definition,

$$\gamma_i = \left| \frac{v_i}{\prod_{j=1}^{n+m} x_j^{f_{i,j}}} \right|_0 \tag{3}$$

The system that results from combining equations 1 and 2 is called Generalized Mass Action (GMA) due to its similarities with Mass Action kinetics.

GMA systems are sometimes approximated by aggregating all the incoming and outgoing fluxes for each metabolite into a single power-law. The equations for the resulting system all have the form:

$$\dot{x}_d = v^+ - v^- = \alpha_i \prod_{j=1}^{n+m} x_j^{g_{i,j}} - \beta_i \prod_{j=1}^{n+m} x_j^{h_{i,j}} \tag{4}$$

The aggregated fluxes can be obtained by writing the stoichiometric matrix as:

$$\mathbf{N} = \mathbf{N}^{+} - \mathbf{N}^{-} \tag{5}$$

where $\mathbf{N}^{+}$ and $\mathbf{N}^{-}$ are matrices containing only the positive and negative entries of $\mathbf{N}$ respectively. Then

$$\begin{aligned} \mathbf{v}^{+} &= \mathbf{N}^{+}\,\mathbf{v} \\ \mathbf{v}^{-} &= \mathbf{N}^{-}\,\mathbf{v} \end{aligned} \tag{6}$$

with this definitions, the matrices of kinetic orders for the s-system, $\mathbf{G}$ and $\mathbf{H}$, can be obtained from that of the GMA, $\mathbf{F}$, using a Taylor series [7] or condensation [4].

$$G = \mathrm{diag}(|\mathbf{v}^{+}|_{\mathbf{0}})\,\mathbf{N}^{+}\,\mathrm{diag}(|\mathbf{v}|_{\mathbf{0}})\,\mathbf{F} \tag{7}$$

$$H = \mathrm{diag}(|\mathbf{v}^{-}|_{\mathbf{0}})\,\mathbf{N}^{-}\,\mathrm{diag}(|\mathbf{v}|_{\mathbf{0}})\,\mathbf{F} \tag{8}$$

where the notation $\mathrm{diag}(\mathbf{x})$ is a square matrix of zeros with vector $\mathbf{x}$ as its main diagonal.So once the flux distribution in the steady state is fixed, the kinetic orders of a GMA can be directly translated to those of an s-system.

1.3 Normalization to the steady state

defining new dependent variables ($\mathbf{z}$) such that $z_i = x_i/|x_i|_0$, and substituting in 2

$$v_i = |v_i|_0 \prod_{j=1}^{n+m} z_j^{f_{i,j}} \tag{9}$$

where $|z_i|_0 = 1\ \forall i$. Applying this normalization to a s-system enables to write the equations of the system in a very convenient form:

$$\dot{\mathbf{z}}_{\mathbf{d}} = \mathrm{diag}(\varphi)\left(\mathbf{z}^{\mathbf{G}} - \mathbf{z}^{\mathbf{H}}\right) \tag{10}$$

after the notation used in [3, 5, 2] where $\mathbf{x}^{\mathbf{A}} = \exp(\mathbf{A}\ \ln(\mathbf{x}))$ and

$$\varphi_i = \left|\frac{v_i^{+}}{x_i}\right|_0 \tag{11}$$

is the turnover of x_i. Thus, knowing the metabolite concentrations, the flux distribution and the matrix of kinetic orders ($\mathbf{F}$) is all the information needed to write down an s-system around such steady state.

1.4 Sensitivity analysis

The response of a power-law system to changes in its parameters (sensitivities) or its independent variables (logarithmic gains) has been thoroughly studied in Biochemical Systems Theory [7] and Metabolic Control Analysis [1]. These sensitivities can be obtained by implicit differentiation of equation 1 but here we will derive them from the s-system form. Both methods reach the same result, but implicit differentiation will involve the metabolite concentrations while calculation from the s-systems is independent of most metabolite concentrations and is often determined exclusively by the kinetic orders.

$$\mathbf{A_D}\,\mathbf{y_D} + \mathbf{A_I}\,\mathbf{y_I} = \mathbf{b} \tag{12}$$

where $\mathbf{A} = \mathbf{G} - \mathbf{H}$ is partitioned into a matrix containing the kinetic orders of the dependent variables $\mathbf{A_D}$ and another for those of the independent variables $\mathbf{A_I}$. and $b_i = \log\beta_i - \log\alpha_i$. When $\mathbf{A_D}$ has full rank, the system has a unique steady state with the analytic solution:

$$\mathbf{y_D} = -\mathbf{A_D}^{-1}\,\mathbf{A_I}\,\mathbf{y_I} + \mathbf{A_D}^{-1}\,\mathbf{b} \tag{13}$$

The calculation of sensitivities to the parameters (rate constants) is straightforward:

$$\boldsymbol{S}(\boldsymbol{x_D},\boldsymbol{\beta}) = -\boldsymbol{S}(\boldsymbol{x_D},\boldsymbol{\alpha}) = \frac{\boldsymbol{\partial}\log\boldsymbol{x_D}}{\boldsymbol{\partial}\log\boldsymbol{\beta}} = \mathbf{A_D}^{-1} \tag{14}$$

and the log gains:

$$\boldsymbol{L}(\boldsymbol{x_D},\boldsymbol{x_I}) = \frac{\boldsymbol{\partial}\log\boldsymbol{x_D}}{\boldsymbol{\partial}\log\boldsymbol{x_I}} = -\mathbf{A_D}^{-1}\,\mathbf{A_I} \tag{15}$$

the sensitivities with respect to the original rate constants ($\boldsymbol{\gamma}$) can be obtained using the chain rule:

$$\left[\frac{\boldsymbol{\partial}\log\boldsymbol{x_D}}{\boldsymbol{\partial}\log\boldsymbol{\gamma}}\right] = \left[\begin{array}{cc}\frac{\boldsymbol{\partial}\log\boldsymbol{x_D}}{\boldsymbol{\partial}\log\boldsymbol{\alpha}} & \frac{\boldsymbol{\partial}\log\boldsymbol{x_d}}{\log\boldsymbol{\beta}}\end{array}\right]\left[\begin{array}{c}\frac{\boldsymbol{\partial}\log\boldsymbol{\alpha}}{\boldsymbol{\partial}\log\boldsymbol{\gamma}}\\ \frac{\log\boldsymbol{\beta}}{\boldsymbol{\partial}\log\boldsymbol{\gamma}}\end{array}\right] \tag{16}$$

$$\boldsymbol{S}(\boldsymbol{x_D},\boldsymbol{\gamma}) = \mathbf{A_D}^{-1}\left[\begin{array}{cc}-\mathrm{diag}(|\boldsymbol{v}^{+}|_{\mathbf{0}}) & \mathrm{diag}(|\boldsymbol{v}^{-}|_{\mathbf{0}})\end{array}\right]\left[\begin{array}{c}\mathbf{N}^{+}\\ \mathbf{N}^{-}\end{array}\right]\mathrm{diag}(|\mathbf{v}|_{\mathbf{0}}) \tag{17}$$

Flux sensitivities of the s-systems are obtained in terms of aggregate fluxes, which means information is lost regarding branch points. This can be avoided by using the GMA flux definition to propagate information from variables to fluxes:

$$\boldsymbol{S}(\boldsymbol{v},\gamma) = \frac{\boldsymbol{\partial} \log \boldsymbol{v}}{\boldsymbol{\partial} \log \gamma} = \mathbf{F_D}\, \boldsymbol{S}(\boldsymbol{x_D},\gamma) + \mathbf{I} \tag{18}$$

and the log gains:

$$\boldsymbol{L}(\boldsymbol{v},\boldsymbol{x_I}) = \frac{\boldsymbol{\partial} \log \boldsymbol{v}}{\boldsymbol{\partial} \log \boldsymbol{x_I}} = \mathbf{F_D}\, \boldsymbol{L}(\boldsymbol{x_D},\boldsymbol{x_I}) + \mathbf{F_I} \tag{19}$$

1.5 Moiety conservations and sensitivity analysis

When there are moiety conservations in a network, the matrix $\mathbf{A_D}$ cannot be inverted the computation of sensitivities involves the so called link matrix [6]. The link matrix is built from the stoichiometric matrix, but it also includes the concentrations of metabolites involved in moiety conservations as fractions of the conservation itself. This creates a difficulty to sample the k- and x-ensembles separately. There are several workarounds to this issue but the most straightforward approach is adding a dummy variable to the k-ensemble for every metabolite involved in a conservation relation. For a network involving NAD and NADH, for instance, two variables, $0 \leq f_{NAD} \leq 1$ and $0 \leq f_{NADH} \leq 1$ such that $f_{NAD} + f_{NADH} = 1$. These variables contain all the information needed to compute the link matrix and it only increase the dimensionality of the k-ensemble in two. If the dummy variables are critical for a given phenotype, the interaction between the ensembles can easily be taken into account.

1.6 Equations for the model used as a case study

The system of equations is set as a GMA-system that due to the simplicity of the pathway also conforms to eq 4

$$\begin{aligned}
\dot{X}_1 &= \gamma_1 X_0^{f_{10}} X_1^{f_{11}} X_3^{f_{13}} - \gamma_2 X_1^{f_{21}} X_2^{f_{22}} \\
\dot{X}_2 &= \gamma_2 X_1^{f_{21}} X_2^{f_{22}} - \gamma_3 X_2^{f_{32}} X_3^{f_{33}} \\
\dot{X}_3 &= \gamma_3 X_2^{f_{32}} X_3^{f_{33}} - \gamma_4 X_3^{f_{43}} X_4
\end{aligned} \tag{20}$$

As explained in the main text, the independent variable X_0 is the concentration of precursor and X_4 is a multiplier introduced to account for increases in demand. Due to the linear nature of the definition of X_4, its kinetic order is fixed at unity.

References

[1] Jan-Hendrik S Hofmeyr. Metabolic control analysis in a nutshell. In *Proceedings of the 2nd International conference on systems biology*, pages 291–300. Omnipress Madison, Wisconsin, 2001.

[2] Hannes Löwe, Andreas Kremling, and Alberto Marin-Sanguino. Time hierarchies and model reduction in canonical non-linear models. *Frontiers in Genetics*, 7:166, 2016.

[3] Alberto Marin-Sanguino, Eduardo R Mendoza, and Eberhard O Voit. Flux duality in nonlinear gma systems: Implications for metabolic engineering. *Journal of biotechnology*, 149(3):166–172, 2010.

[4] Alberto Marin-Sanguino, Eberhard O Voit, Carlos Gonzalez-Alcon, and Nestor V Torres. Optimization of biotechnological systems through geometric programming. *Theoretical Biology and Medical Modelling*, 4(1):38, 2007.

[5] Stefan Müller and Georg Regensburger. Generalized mass-action systems and positive solutions of polynomial equations with real and symbolic exponents (invited talk). In *International Workshop on Computer Algebra in Scientific Computing*, pages 302–323. Springer, 2014.

[6] Christine Reder. Metabolic control theory: a structural approach. *Journal of theoretical biology*, 135(2):175–201, 1988.

[7] Michael A Savageau. *Biochemical systems analysis. A study of function and design in molecular biology.* 1976.
